# *Perk* Gene Dosage Regulates Glucose Homeostasis by Modulating Pancreatic β-Cell Functions

**DOI:** 10.1371/journal.pone.0099684

**Published:** 2014-06-10

**Authors:** Rong Wang, Elyse E. Munoz, Siying Zhu, Barbara C. McGrath, Douglas R. Cavener

**Affiliations:** The Pennsylvania State University, Department of Biology, Center of Cellular Dynamics, University Park, Pennsylvania, United States of America; NIDCR/NIH, United States of America

## Abstract

**Background:**

Insulin synthesis and cell proliferation are under tight regulation in pancreatic β-cells to maintain glucose homeostasis. Dysfunction in either aspect leads to development of diabetes. PERK (EIF2AK3) loss of function mutations in humans and mice exhibit permanent neonatal diabetes that is characterized by insufficient β-cell mass and reduced proinsulin trafficking and insulin secretion. Unexpectedly, we found that *Perk* heterozygous mice displayed lower blood glucose levels.

**Methodology:**

Longitudinal studies were conducted to assess serum glucose and insulin, intracellular insulin synthesis and storage, insulin secretion, and β-cell proliferation in *Perk* heterozygous mice. In addition, modulation of *Perk* dosage specifically in β-cells showed that the glucose homeostasis phenotype of *Perk* heterozygous mice is determined by reduced expression of PERK in the β-cells.

**Principal Findings:**

We found that *Perk* heterozygous mice first exhibited enhanced insulin synthesis and secretion during neonatal and juvenile development followed by enhanced β-cell proliferation and a substantial increase in β-cell mass at the adult stage. These differences are not likely to entail the well-known function of PERK to regulate the ER stress response in cultured cells as several markers for ER stress were not differentially expressed in *Perk* heterozygous mice.

**Conclusions:**

In addition to the essential functions of PERK in β-cells as revealed by severely diabetic phenotype in humans and mice completely deficient for PERK, reducing *Perk* gene expression by half showed that intermediate levels of PERK have a profound impact on β-cell functions and glucose homeostasis. These results suggest that an optimal level of PERK expression is necessary to balance several parameters of β-cell function and growth in order to achieve normoglycemia.

## Introduction

The endocrine pancreatic β-cells have an exclusive and singular function to synthesize and secrete insulin. While insulin is essential for maintaining glucose homeostasis, hyperinsulinemia can result in hypoglycemic shock and death. Therefore insulin synthesis and secretion must be tightly regulated to provide the appropriate level of circulating insulin in response to episodic input of dietary carbohydrates and release of glucose stores. Pancreatic insulin output is controlled by a combination of regulating β-cell mass in the endocrine pancreas [1]–[4] and by regulating insulin synthesis and secretion in β-cells [5]–[9]. Although a large number of genes have been shown to influence β-cell growth and insulin synthesis and secretion, a small number of genes (ca. 20) including *Perk* have been identified in humans that are absolutely essential for β-cell growth or insulin production [10], [11]. The consequence of the loss of function mutations in these genes is permanent neonatal diabetes (PND). Among these PND genes, the function of the *Perk* (EIF2AK3) gene has been the most controversial and perplexing [12]–[15]. *Perk* was initially identified as one of the three regulatory arms of the ER stress response pathway in cultured mammalian cells [16], [17]. Shortly after its discovery [18] and characterization in cell culture, mutations in *Perk* were found to be the cause of the Wolcott-Rallison syndrome (WRS) in humans [19] that featured permanent neonatal diabetes, exocrine pancreas deficiency, growth retardation, and osteopenia. *Perk* knockout (KO) mouse strains were generated by us [15] and by Harding and Ron [12], which exhibited a nearly identical phenotype to that seen in human WRS patients, including permanent neonatal diabetes. By generating and analyzing tissue-specific *Perk* KO and transgenic rescue strains, we showed that the neonatal diabetes was caused by deficient β-cell growth and multiple problems in proinsulin synthesis and trafficking and insulin secretion [13], [14], [20].

An extensive analysis of PERK function by us has failed to support the initial hypothesis that the β-cell defects seen in *Perk* deficiency are due to misregulation of the ER stress response pathway [13], [14]. Moreover, mutations in the other two regulatory arms of the ER stress pathway, ATF6 and IRE1, do not cause major β-cell dysfunctions or diabetes [21], [22]. This demonstrates that dysfunction in the ER stress response generally does not result in permanent neonatal diabetes. Some of these β-cell dysfunctions seen in *Perk* KO mice can be attributed to the lack of phosphorylation of eIF2α, the primary substrate of PERK, because mutations that block the Ser51 phosphorylation site either in whole animals or in just the β-cells also result in diabetes [23], [24]. However, other PERK-dependent β-cell functions may be independent of eIF2α phosphorylation including regulation of secretagogue stimulated calcium influx and insulin secretion [25].

Humans and mice that are heterozygous for a loss-of-function *Perk* mutation do not exhibit overt abnormal phenotypes[15], [19], [26]. However, we found that *Perk* heterozygous (*Perk^+/−^*) mice exhibit significantly lower serum blood glucose levels among several hundred litters of mice analyzed over the past ten years, opposite to the *Perk* KO mice which are severely hyperglycemic. To determine the underlying reasons for this shift in glucose homeostasis of *Perk^+/−^* mice, we conducted a postnatal developmental analysis of β-cell growth and function in *Perk^+/−^* mice compared to their homozygous wild-type littermates. We found that *Perk^+/−^* mice first exhibited enhanced insulin synthesis and secretion during neonatal and juvenile development followed later at the adult stage by enhanced β-cell proliferation and a substantial increase in β-cell mass. These findings support the hypothesis that PERK dynamically regulates β-cell growth, insulin synthesis and secretion during postnatal development.

## Methods and Materials

### Genetic Strains

*Perk* global KO allele, floxed *Perk* allele were generated as previously described [15]. A Perk transgene under the control of the rat insulin promoter was introduced into the wild-type mice to generate mice with overexpression of *Perk* specifically in β-cells with an otherwise wild-type background (*Perk^+/+^;βPerk*) [27]. *Perk^+/−^* mice that carried one *Perk* KO allele were in congenic C57Bl/6, 129 SvEvTac, or in a mixed background. To generate pancreatic specific *Perk^+/−^* mice, *pdx1-cre* transgenic mice were crossed with mice homozygous for floxed *Perk* allele. Mice were sacrificed by CO_2_ euthanasia. All animal studies were approved by the Institutional Animal Care and Use Committee of Pennsylvania State University, and all efforts were made to minimize suffering.

### Islet isolation

Mouse islets were isolated using a modified Histopaque-1077 separation method [28]. Before experiments, islets were allowed overnight recovery in fresh RPMI1640 medium with 10% fetal bovine serum, 1% Antibiotic Antimycotic Solution (Sigma) and 5.5 mM glucose at 5% CO_2_, 95% air.

### Cell culture

*INS1* 832/13 cells containing a short-hairpin RNA directed against the rat *Perk* mRNA (*shPerk*) were obtained from Dr. Fumihiko Urano (University of Massachusetts). The *shPerk* is stably integrated into the genome of *INS1* 832/13 β-cell lines and under the inducible regulation of doxycycline. The *INS1* 832/13 *shPerk* cells were cultured in a tetracycline-free environment to avoid leaky expression of *shPerk*.

### Determination of serum glucose and glucose tolerance test

Blood samples were obtained from the tails and glucose were measured using OneTouch Ultra glucose meters. Glucose tolerance tests were performed on mice fasted 4 hours (for P17 mice) or overnight (for P50 mice) and injected intraperitoneally with 2 mg glucose/g of body weight.

### Insulin measurement

Insulin concentrations were determined by immunoassay (Meso Scale Discovery, MSD). For serum insulin measurement, serum was obtained by centrifugation of blood samples at 10,000 g for 5 min. For islet and pancreatic insulin measurement, islets or pancreata were sonicated in 1 ml of cold acid ethanol (1.5% volume HCl in 75% ethanol). Insulin concentrations were further normalized to total protein concentration (determined by BIO-RAD Protein Assay). For studies of glucose stimulated insulin secretion, isolated islets or cultured β-cell line were firstly cultured overnight at 37°C (5% CO_2_) in RPMI1640 medium containing 10% fetal bovine serum and 5.5 mM glucose. Samples were then incubated at 37°C in KRB-HEPES buffer (pH 7.4) with 1% bovine serum albumin and 2.8 mM glucose for 1 hour before insulin stimulation with 2.8 or 20 mM glucose. At the end of the 30 min stimulation, the supernatant was assayed for secreted insulin (by MSD), and cells/islets were assayed for total insulin and total protein.

### RNA isolation and gene expression measurement

RNA was extracted from pancreas, islets or cultured cells using RNeasy Mini Kit (Qiagen) and quantified by RiboGreen RNA Assay Kit (Invitrogen). Reverse transcription was performed using qScript cDNA supermix (Quanta). Quantitative mRNA measurement was carried out by using qPCR core kit for SYBR Green I (Quanta) with the StepOne Plus detection system (Applied Biosystems). Levels of Xbp-s (spliced form) were normalized to Xbp-t (total) levels. Other gene expression levels were normalized to Gapdh and Actin levels of the same sample. Mouse primer sequences were listed as follows:

*Actin 5′-GCCCTGAGGCTCTTTTCC-3′, 5′-TGCCACAGGATTCCATACCC-3′;*

*Gapdh 5′-GGAGCGAGACCCCACTAACA-3′, 5′-ACATACTCAGCACCGGCCTC-3′;*

*Perk, 5′-TCCTGCTTTGCATCGTAGCC-3′, 5′-GATGGAAAAGCCTGCGCA-3′;*

*Insulin II exon, 5′-CAGAAGCGTGGCATTGTAGA-3′, 5′-TTGCAGTAGTTCTCCAGCTGG-3′; Insulin II intron, 5′-CCTAGGTGTGGAGGGTCTCG-3′, 5′-CCAGAAACGTGTCCCCACTC-3′; glucagon, 5′- GAACAACATTGCCAAACGTCA-3′, 5′-GTCCCTTCAGCATGCCTCTC-3′;*

*MafA, 5′-GCTGGTATCCATGTCCGTGC-3′, 5′-GTCGGATGACCTCCTCCTTG-3′;*

*Pdx-1, 5′-GAGCGTTCCAATACGGACCA-3′,5′-TCAGCCGTTCTGTTTCTGGG-3′;*

*Hrd1, 5′-TGGCTTTGAGTACGCCATTCT-3′, 5′-CCACGGAGTGCAGCACATAC-3′;*

*ERp57, 5′-GGCGGATGCAACATATCACC-3′, 5′-TGTGGTTCGTACTGTCCCCC-3′;*

*BiP, 5′-GCTTCGTGTCTCCTCCTGAC-3′, 5′-TAGGAGTCCAGCAACAGGCT-3′;*

*ERp72, 5′-TTCCACGTGATGGATGTTCAG-3′, 5′-AGTCTTACGATGGCCCACCA-3′;*

*Chop, 5′-CCAACAGAGGTCACACGCAC-3′, 5′-TGACTGGAATCTGGAGAGCGA-3′;*

*Atf4, 5′-GAG TTT GAC TTC GAT GCT CTG TTT C-3′, 5′-TGG CAT GGT TTC CAG GTC AT-3′; Xbp1-spliced, 5′-GAGTCCGCAGCAGGTG-3′, 5′-GTGTCAGAGTCCATGGGA-3′;*

*Xbp1-total, 5′-CACCTTCTTGCCTGCTGGAC-3′, 5′-GGGAGCCCTCATATCCACAGT-3′.*

*Rat primer sequences were listed as follows:*

*Actin, 5′-ATC CTG GCC TCA CTG TCC AC-3′, 5′-CTA GAA GCA TTT GCG GTG CA-3′; Gapdh, 5′-CACCACCAACTGCTTAGCCC-3′, 5′-TGGCATGGACTGTGGTCATG-3′;*

*Perk, 5′-TTGGGCTAGTGACTGCAATG-3′, 5′-TTGTCCCGTGTGTGTAGCAT-3′;*

*Insulin I, 5′-CAGCACCTTTGTGGTCCTCA-3′, 5′-CCCACACACCAGGTACAGAGC-3′;*

*Insulin II, 5′-CTGCCCAGGCTTTTGTCAAA-3′, 5′-CTTCCACCAAGTGAGAACCACA-3′;*

*ERp72, 5′-TCTAACCAATCACCGGGCTG-3′,5′-TCATGGTAAGGTGCCGAGG-3′.*

### Western Blot Analysis

Total cellular protein was extracted from 60 islets or 10^6^ cells per sample with RIPA buffer (1% Nonidet P40, 0.5% sodium doxycholate, 0.1% SDS, 1 × PBS, pH 8.0) containing 1×protease and phosphatase inhibitor cocktails (Sigma) and were boiled in 2×SDS sample buffer and then loaded onto 4–15% gels for Western blots. To measure newly synthesized proinsulin the SUnSET method was used [29]. Briefly, 10 µg/ml puromycin was added 15 minutes before harvest. Western blot was performed and proteins were probed with mouse anti-puromycin (1∶5000, Millipore) and rabbit anti-proinsulin C-peptide (1∶500, Bio Vision) primary antibodies, followed by IR800 anti-mouse and IR700 anti-rabbit secondary antibodies (1∶20000, LI-COR) for visualization of newly synthesized proteins co-localized with proinsulin using LI-COR Odyssey scanner. Other primary antibodies used in western blot analysis were: anti-PERK (1∶500, Cell Signaling), anti-ERp72 (1∶1000, Stressgen, Inc), anti-GRP78/BiP (1∶500, Santa Cruz, Inc.), anti-ERp57 (1∶500, Santa Cruz), anti-PDI (1∶500, Stressgen, Inc.).

### Measurement of β-cell proliferation and volume

To measure β-cell proliferation, all experimental mice received BrdU at a concentration of 0.8 mg/ml in drinking water. Mice ≤ P17 days were treated with BrdU water for 3 days, and mice ≥ 30 days were treated for 7 days. After BrdU administration, whole pancreata were harvested for immunohistochemistry following the standard procedure as previously described [30]. Guinea pig Anti-insulin (1∶500, Abcam) and mouse anti-BrdU (1∶50, DAKO) were applied overnight at 4°C. Anti-guinea pig and anti-mouse secondary antibodies conjugated with Alexa Fluor488 and 555 dye (Molecular Probes) were used (1∶400 dilution) to visualize the labeled cells. Anti-fade reagent with Dapi (Life technologies) was used to mount slides and label the nucleus. Fluorescence images were captured with a Nikon Eclipse E1000 and Image-Pro Plus (Phase 3 Imaging Systems, GE Healthcare, Inc.). To calculate β-cell daily proliferation, ratios of BrdU-positive to total β-cells were counted from a total of 4 tissue sections per mouse and further divided by total days of BrdU treatment. The same images were used for β-cell volume estimates. Insulin-positive cells were traced by using imageJ software, and single cell volume was estimated by dividing the total area of β-cells by cell number.

### Estimation of β-cell number

Total β-cell number was estimated by a method we used previously [14], [31]. The total amount of *Glut2* mRNA or *Insulin II* mRNA in whole pancreata was first determined and then divided by the estimated amount of Glut2 or Insulin II mRNA, respectively, per β-cell. Since these two genes are exclusively expressed in β-cells of the endocrine pancreatic islets, the ratio of their total pancreatic level to single cell level can be used to estimate total β-cell number [14], [31]. Similarly, β-cell number was estimated by dividing insulin protein level in whole pancreata by insulin content per β-cell.

### Statistical Analysis

All numerical data were represented as mean ± SE. Statistical significance was determined using Student's *t* testing.

## Results

### *Perk* heterozygous mice exhibit increased levels of circulating insulin and decreased glucose

**Figure 1:**
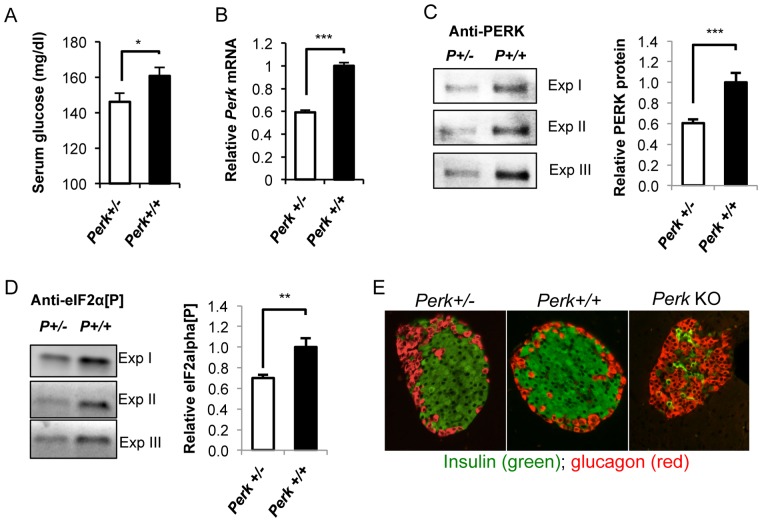
Modulation of *Perk* dosage using *Perk^+/−^* mice impacted glucose homeostasis. A. Random fed serum glucose of mice at 15–36 postnatal days. Mice are in mixed genetic background. Shown are means ± SEM (n = 44, 68; *P<0.05). B. *Perk* mRNA measurement in mouse islets expressed as percentage of *Perk^+/+^* levels. Shown are means ± SEM (n = 29, 26; ***P<0.001). C. Western blot of PERK protein in mouse islets. Left panel showed representative blots. Right panel showed quantification of PERK protein levels in relative to *Perk^+/+^*. (n = 5, 5; ***P<0.001). D. Western blot of phosphorylated eIF2α in mouse islets. Left panel showed representative blots. Right panel showed quantification of PERK protein levels in relative to *Perk^+/+^*. (n = 5, 4; **P<0.01). E. Immuno-staining of pancreatic section from *Perk^+/−^*, *Perk^+/+^*, and *Perk* KO mice using insulin and glucagon antibody.

Mice and humans that are completely deficient for PERK exhibit severe hyperglycemia (>400 mg/dl) even in the fasted state [12], [14], [15], [19], [26]. Therefore we expected that half dosage of PERK associated with *Perk* heterozygosity would either be recessive with no effect on glucose homeostasis or would be semi-dominant with elevated blood glucose. Surprisingly we found that adult *Perk^+/−^* mice exhibited significantly lower random-fed serum glucose levels than mice homozygous for the normal (*Perk^+/+^)* wild-type allele (Fig 1A). The mouse *Perk* KO allele used in this study was generated by us [15] and utilized in several previous studies [13]–[15], [20], [25], [27], [30], [32]–[37]. Our *Perk* KO allele was made by Cre-mediated deletion of exons 5–7 that encoded part of the luminal domain, the transmembrane domain, and part of the catalytic domain of PERK yielding a loss-of-function null allele. Consistent with this, *Perk^+/−^* mice exhibited the expected reduction in *Perk* mRNA and protein (ca. 60% normal, P<0.001) (Fig 1B–C) levels compared to wild-type *Perk^+/+^* mice. Moreover, reduction in PERK activity was observed in *Perk^+/−^* β-cells as phosphorylation of eIF2α, a downstream substrate of PERK, was significantly reduced by 30% (P<0.01) (Fig 1D). Although circulating glucose levels and PERK activity are reduced, the islet composition and β-cell morphology in the pancreata of *Perk^+/−^* is indistinguishable from *Perk^+/+^* and these observations are in contrast to radical changes seen *Perk* KO mice, which include reduced β-cell number (Fig 1E). *Perk^+/−^* β-cells also show normal enrichment of proinsulin in the Golgi (not shown) in contrast to ER retention seen *Perk* KO β-cells [38].

**Figure 2:**
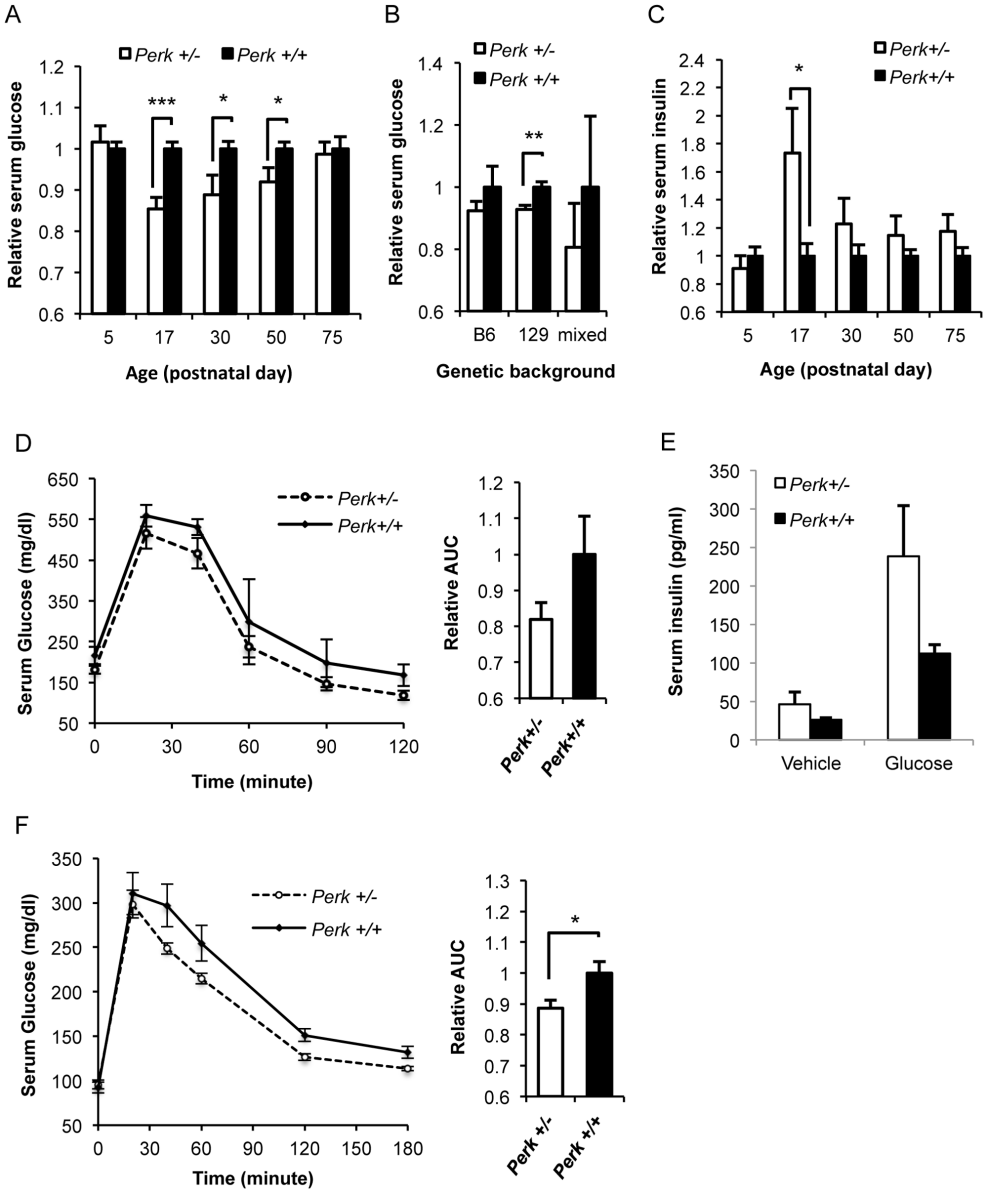
Glucose and insulin homeostasis were impacted by modulation of *Perk* during development. A. Random fed serum glucose of *Perk^+/−^* mice relative to *Perk^+/+^* during postnatal development. Age of mice was shown as indicated, except that mice belonging to P75 group were from P70 to P90. Mice are in C57BL/6 background. Shown are means ± SEM (n for *Perk ^+/−^, Perk^+/+^*  =  18, 16 (P5); 16, 17 (P17); 13, 11 (P30); 12, 16 (P50); 17, 18 (P75). *P<0.05, ***P<0.001). B. Random fed serum glucose of *Perk^+/−^* mice relative to *Perk^+/+^* at 9–12 month. Mice are in C57BL/6, 129 SvEvTac or mixed background. Shown are means ± SEM. (n for *Perk ^+/−^, Perk^+/+^*  =  4, 5 (B6); 23, 20 (129); 5, 3. **P<0.01). C. Random fed serum insulin of *Perk^+/−^* mice relative to *Perk^+/+^* during postnatal development. Age of mice was shown as indicated, except that mice belonging to P75 group were from P70 to P90. Shown are means ± SEM (n for *Perk ^+/−^, Perk^+/+^*  =  28, 28 (P5); 19, 17 (P17); 25, 18 (P30); 56, 75 (P50); 29, 32 (P75). *P<0.05). D. Glucose tolerance test of P17 mice after 4 hour fasting. Serum glucose was measured at various time points after glucose injection as indicated above. GTT traces are shown as means ± SEM of each time point and quantified by calculating area under the curve (AUC) in relative to *Perk^+/+^* (n for *Perk ^+/−^, Perk^+/+^*  = 5, 4). E. Serum insulin collected in P17 mice 30 minutes after injection with glucose or vehicle (n for *Perk ^+/−^, Perk^+/+^*  = 5, 4. P = 0.16 for stimulated insulin levels). F. Glucose tolerance test of P50 mice after 16 hour fasting. Experiment and analysis were as in 2D (n for *Perk ^+/−^, Perk^+/+^*  = 18, 18, *P<0.05).

To determine when in development *Perk^+/−^* mice first display mild hypoglycemia, we analyzed a large cohort of mice at various postnatal stages (Fig 2A). The genetic background of these mice was congenic C57Bl/6. *Perk^+/−^* and *Perk^+/+^* littermates were used throughout this study to reduce inter-litter variation. Blood glucose levels of neonatal (postnatal day 5) *Perk^+/−^* mice were indistinguishable compared to *Perk^+/+^*, but as mice approached the weaning stage of development (postnatal day 17) a substantial reduction in blood glucose levels was seen in *Perk^+/−^* mice. Curiously, the difference in blood glucose between *Perk^+/−^* and *Perk^+/+^* mice was reduced with age. By the time the mice reached postnatal 75 days, the difference in blood glucose was no longer statistically significant. However, male *Perk^+/−^* mice showed a persistent trend towards reduced blood glucose as seen in 9–12 month old mice in three genetic backgrounds (Fig 2B) including a highly statistically significant difference for *Perk^+/−^* in a 129 SvEvTac genetic background (Fig 2B, P<0.01). Serum insulin levels were inversely proportional to serum glucose levels with *Perk^+/−^* mice (C57Bl/6 genetic background) showing significantly higher levels than *Perk^+/+^* at P17 (P<0.05, Fig 2C) and a non-significant trend towards higher insulin levels in older mice.

To determine if *Perk* dosage would impact serum glucose and insulin under glucose stimulation, a glucose tolerance test (GTT) was performed in juvenile (P17) and adult mice (P50). *Perk^+/−^* mice trended towards being more glucose tolerant (P = 0.1) than WT in P17 mice (Fig 2D) and were significantly more glucose tolerant in P50 mice (P<0.05, Fig 2F). Serum insulin exhibited a strong trend towards being higher in *Perk^+/−^* mice injected with glucose (Fig 2E) but was not quite statistically significant.

### Insulin content and β-cell are modulated by *Perk* gene dosage during postnatal development

**Figure 3:**
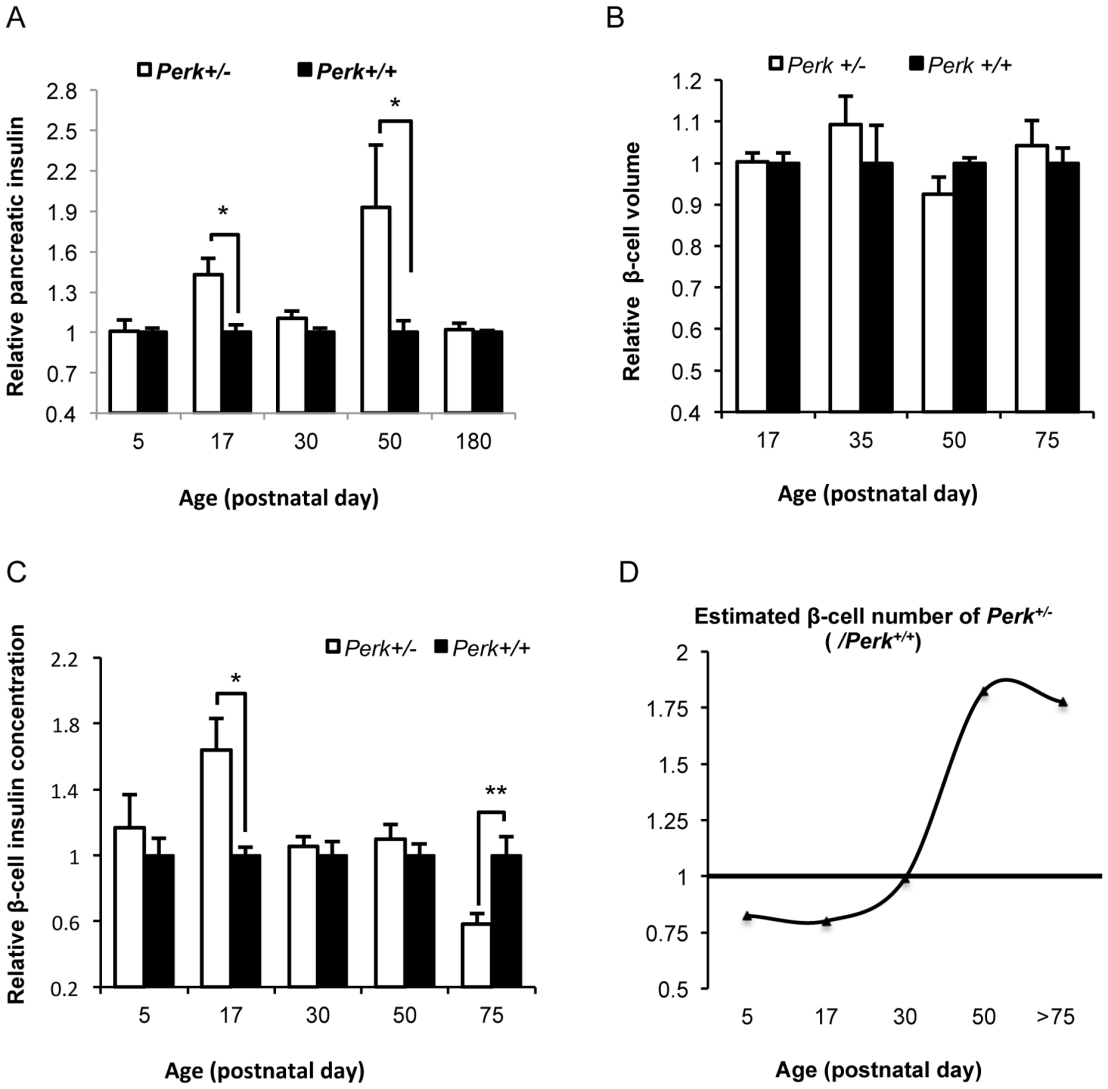
Insulin production was modulated by *Perk* during development. A. Total pancreatic insulin content of *Perk^+/−^* mice relative to *Perk^+/+^* during postnatal development. Shown are means ± SEM (n for *Perk ^+/−^, Perk^+/+^*  =  14, 16 (P5); 8,5 (P17); 18, 16 (P30); 10, 15 (P50); 8, 6 (P180). *P<0.05). B. β-cell volume of *Perk^+/−^* mice relative to *Perk^+/+^* during postnatal development. Shown are means ± SEM (n for *Perk ^+/−^, Perk^+/+^*  =  3, 4 (P17); 4, 4 (P35); 4, 3 (P50); 3, 3(P75). No statistical significance was observed at any developmental time point). C. Estimated β-cell insulin concentration of *Perk^+/−^* mice relative to *Perk^+/+^* during postnatal development. Age of mice was shown as indicated, except that mice belonging to P75 group had ages ranging from P70 to P90. Shown are means ± SEM (n for *Perk ^+/−^, Perk^+/+^*  =  7,6 (P5); 9, 11 (P17); 6, 9 (P30); 11, 16 (P50); 9, 8(P75). *P<0.05, **P<0.01). D. Estimated β-cell number of *Perk^+/−^* mice relative to *Perk^+/+^* using insulin content in the whole pancreas divided by estimated insulin content per cell.

To determine whether insulin synthesis and storage contribute to PERK-dependent regulation of glucose and insulin homeostasis, whole pancreatic insulin content was measured. Compared to *Perk^+/+^*, *Perk^+/−^* mice exhibited 42.8% and 92.8% increase of pancreatic insulin at P17 and P50 (P<0.05 at both ages) (Fig 3A), respectively. By contrast, there was no *Perk*-dependent difference at other developmental time points, suggesting a developmental complexity underlying the *Perk* genotype differences in glucose homeostasis. These observed differences in total pancreatic insulin could arise from either differences in insulin content per β-cell or differences in total β-cell number. We estimated cell volume and insulin concentration per β-cell, both of which determine the insulin content per β-cell. β-cell volume did not differ between genotypes at any developmental stage (Fig 3B), suggesting that *Perk* genotypic difference in insulin content is due to a difference in β-cell insulin concentration. We used the concentration of insulin per islet cells as an estimate of the insulin concentration per β-cell, given the fact that β-cells comprise most of the islet mass and that cell-type composition of islets is not different between genotypes (data not shown). At P17, β-cell insulin concentration was significantly higher in *Perk^+/−^* compared to *Perk^+/+^* (P<0.05, Fig 3C), suggesting that the elevated whole pancreatic insulin content at P17 was due to increased cellular insulin production and/or storage. The per-β-cell insulin concentration was equivalent in *Perk* genotypes at P5, P30, and P50. Interestingly, by contrast to juvenile mice, 6-month old (P180) *Perk^+/−^* mice showed significantly reduced insulin concentration per β-cell revealing an unexpected developmental complexity. We also estimated pancreatic β-cell number by dividing the total amount of insulin in whole pancreata by the estimated insulin content per β-cell. We found that *Perk^+/−^* mice initially had fewer β-cells during neonatal development, but this trend was reversed in mature adult mice (Fig 3D). In summary, neonatal *Perk^+/−^* mice exhibit higher insulin content per β-cell, enhanced insulin synthesis but fewer β-cells, whereas in mature adult *Perk^+/−^* mice insulin concentration is reduced, while β-cell number is increased.

### β-cell number is increased in *Perk* heterozygotes due to elevated β-cell proliferation

**Figure 4:**
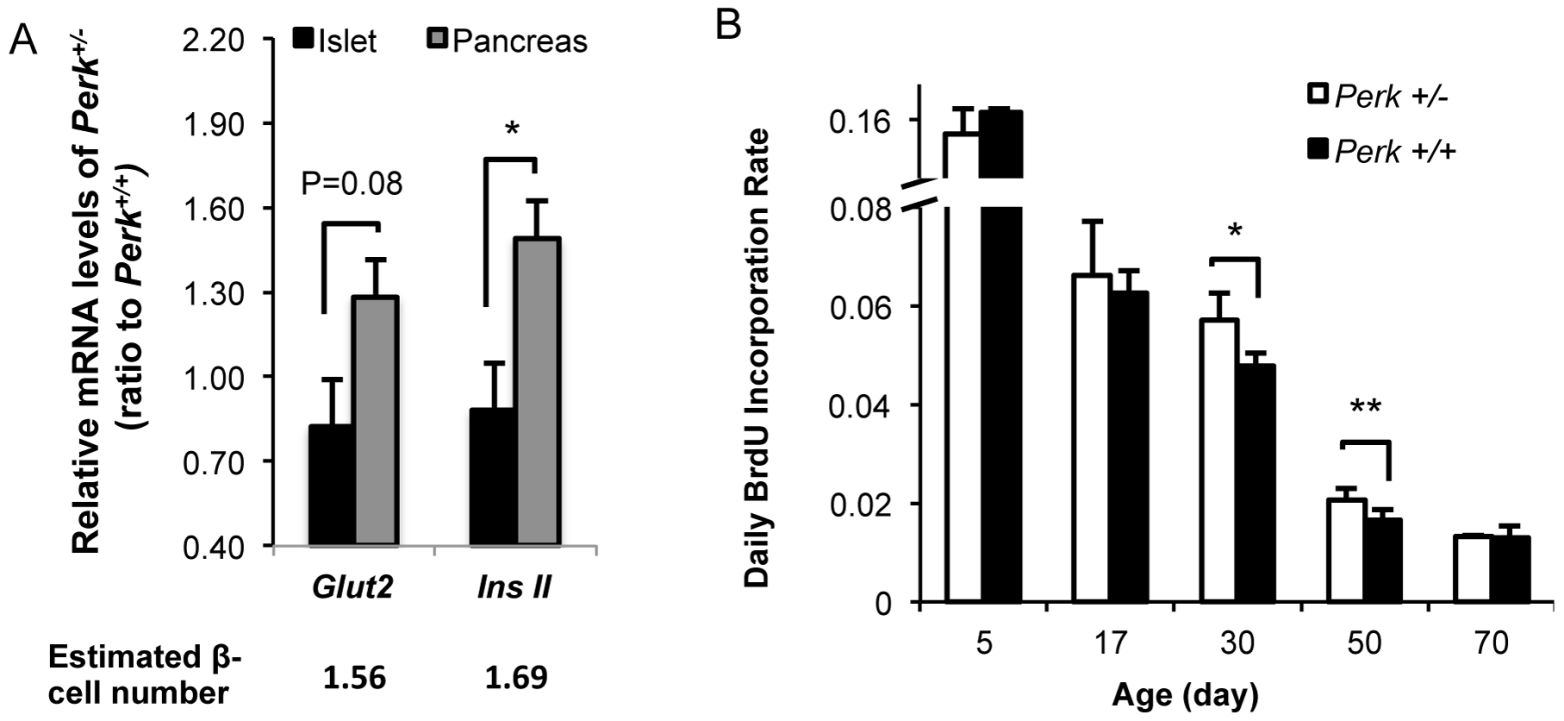
P50 *Perk^+/−^* mice exhibit higher β-cell number due to elevated β-cell proliferation. A. Gene expression levels of islets or whole pancreata from P50 *Perk^+/−^* mice relative to *Perk^+/+^* control. Shown are means ± SEM. Below the bar graph shows the estimated fold-increase of β-cell number of P50 *Perk^+/−^* mice relative to *Perk^+/+^* control using each β-cell specific gene (islet data: n = 3, 4; pancreas data: n = 6, 4. *P<0.05). B. Daily BrdU incorporation rate of β-cells in mice at various ages. Shown are means ± SEM (n for *Perk ^+/−^, Perk^+/+^*  =  5, 5 (P5); 3, 4 (P17); 10, 10 (P30); 4, 4 (P50); 3, 3 (P70). *P<0.05).

Unlike P17 mice, *Perk^+/−^* mice at P50 did not show increased expression of *insulin* mRNA level (Fig 4B) or protein level per β-cell (Fig 3C). However, P50 *Perk^+/−^* mice had substantially higher β-cell number (Fig 3D). To confirm this observation, β-cell number was estimated using the expression of mRNA of two genes, *insulin II* and *Glut2*, after previously published methods [38], [39]. Since both genes are exclusively expressed in β-cells, their mRNA levels in whole pancreata are directly proportional to β-cell number [38], [39]. *Perk^+/−^* mice at P50 had higher total *insulin* (P<0.05, Fig 4A) and total *Glut2* (p = 0.08) mRNA in the total pancreas compared to wild-type mice whereas expression levels of these two genes in islets were not different between genotypes (Fig 4A), reflecting a 56%–69% increase in total β-cells in *Perk^+/−^* (Fig 4A) with equivalent level of expression of *insulin II and Glut2* per β-cell.

To further investigate the reason for increased β-cell number in P50 *Perk^+/−^* mice, β-cell proliferation was determined by BrdU incorporation. β-cell proliferation was found to be significantly increased in P50 *Perk^+/−^* mice compared to WT controls (Fig 4B). We also examined β-cell proliferation at four other developmental time points and found that *Perk^+/−^* exhibited elevated proliferation at P30 and P50 but not earlier or later time points (Fig 4B), indicating that enhanced proliferation was transient and corresponded to the time period when β-cell number was increased in *Perk^+/−^* mice. In addition, β-cell death was estimated using TUNEL assay and found to be negligible and not different between *Perk* genotypes (data now shown).

### Insulin transcription and proinsulin synthesis were up-regulated in *Perk+/−* mice at postnatal day 17

**Figure 5:**
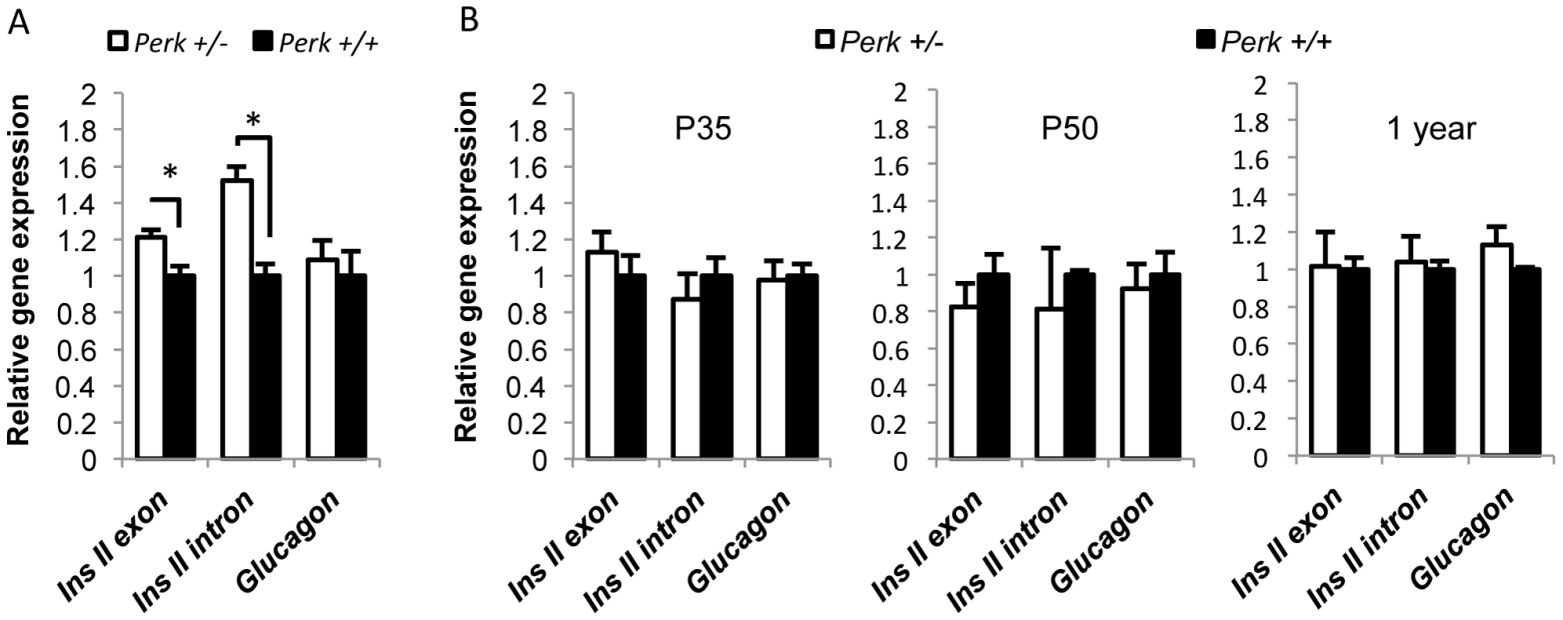
Insulin transcription was up-regulated in P17 *Perk^+/−^* mice. A. Gene expression levels of mouse islets relative to *Perk^+/+^*. Mice are postnatal 17 days. Shown are means ± SEM. (n for *Perk ^+/−^, Perk^+/+^*  = 11,9. *P<0.05). B. Gene expression level of mouse islets relative to *Perk^+/+^*. Islets were isolated from mice at various ages as indicated. Shown are means ± SEM. (n for *Perk ^+/−^, Perk^+/+^*  =  8, 8 (P35); 3, 4 (P50); 6, 6(1 year). No significant difference was seen for any gene at any age).

Despite a lower number of β-cells, P17 *Perk^+/−^* mice exhibited higher pancreatic insulin due to a significant increase in insulin content per β-cell (Fig 3A–D). To probe the mechanism underlying the increased β-cell insulin content in *Perk^+/−^* P17 mice, *Insulin* mRNA was measured in *Perk^+/−^* islets and found to be 21% higher (P<0.05) than WT. To determine if increased *Insulin* gene transcription was responsible for the increased steady-state levels of *Insulin* mRNA, *Insulin* pre-mRNA, which has a much shorter half-life and is less abundant than the mature mRNA, was measured using primers detecting the *Insulin* intron after methods of Evans-Molina and coworkers [40]. *Insulin* pre-mRNA was elevated 52% in *Perk^+/−^* β-cells (P<0.05), whereas *Glucagon* mRNA was not impacted by modulation of *Perk* (Fig 5A), suggesting that *Perk*-dependent difference in *Insulin* gene transcription contributed to the difference in insulin content. Unlike stage P17 mice, no change of mature mRNA or pre-mRNA of insulin was seen in *Perk^+/−^* β-cells at other developmental time points (Fig 5B).

**Figure 6:**
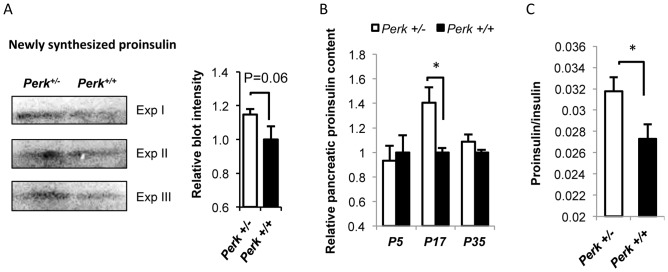
New proinsulin synthesis and mature proinsulin level were up-regulated in P17 *Perk^+/−^* mice. A. Newly synthesized proinsulin in mouse islets measured by western blot. Mice are postnatal 17 days. 10 µg/ml puromycin was added 15 minutes before protein samples were harvested. Left panel showed representative blots probed with anti-puromycin and co-localized with proinsulin C-peptide. Right panel showed quantification of blot intensity relative to *Perk^+/+^*. (n for *Perk ^+/−^, Perk^+/+^*  = 5, 4). B. Total pancreatic proinsulin content of *Perk^+/−^* mice relative to *Perk^+/+^* at various postnatal stages. Shown are means ± SEM (n for *Perk ^+/−^, Perk^+/+^*  =  8, 8 (P5); 8,7 (P17); 18, 17 (P30). *P<0.05). C. Proinsulin and insulin were measured in the pancreatic samples harvested from P17 mice and proinsulin/insulin ratio was calculated. Shown are means ± SEM (n for *Perk ^+/−^, Perk^+/+^*  = 8,7. *P<0.05).

To determine if proinsulin protein synthesis in *Perk^+/−^* at P17 reflected the higher levels of insulin mRNA, new proinsulin synthesis was estimated by puromycin incorporation [29]. *Perk^+/−^* exhibited a 15% higher proinsulin synthesis (Fig 6A), which was nearly statistically significant (p = 0.06). Consistently, pancreatic mature proinsulin was found to be 41% higher in P17 *Perk^+/−^* mice (P<0.05) than *Perk^+/+^* mice (Fig 6B). In contrast, no genotypic difference in mature proinsulin level was observed in mice at other developmental time points (Fig 6B). Moreover, proinsulin to insulin ratio was significantly elevated in P17 *Perk^+/−^* mice (Fig 6C). Taken together, our data suggest that the increased insulin content per β∼cell seen in the *Perk^+/−^* mice at P17 is the result of an increase in all aspects of insulin biosynthesis, including insulin gene transcription, proinsulin synthesis and maturation. It is also possible that genotypic differences in proinsulin and/or insulin stability could contribute to the observed differences in proinsulin and insulin.

### ER chaperones are differentially regulated by *Perk* gene dosage

**Figure 7:**
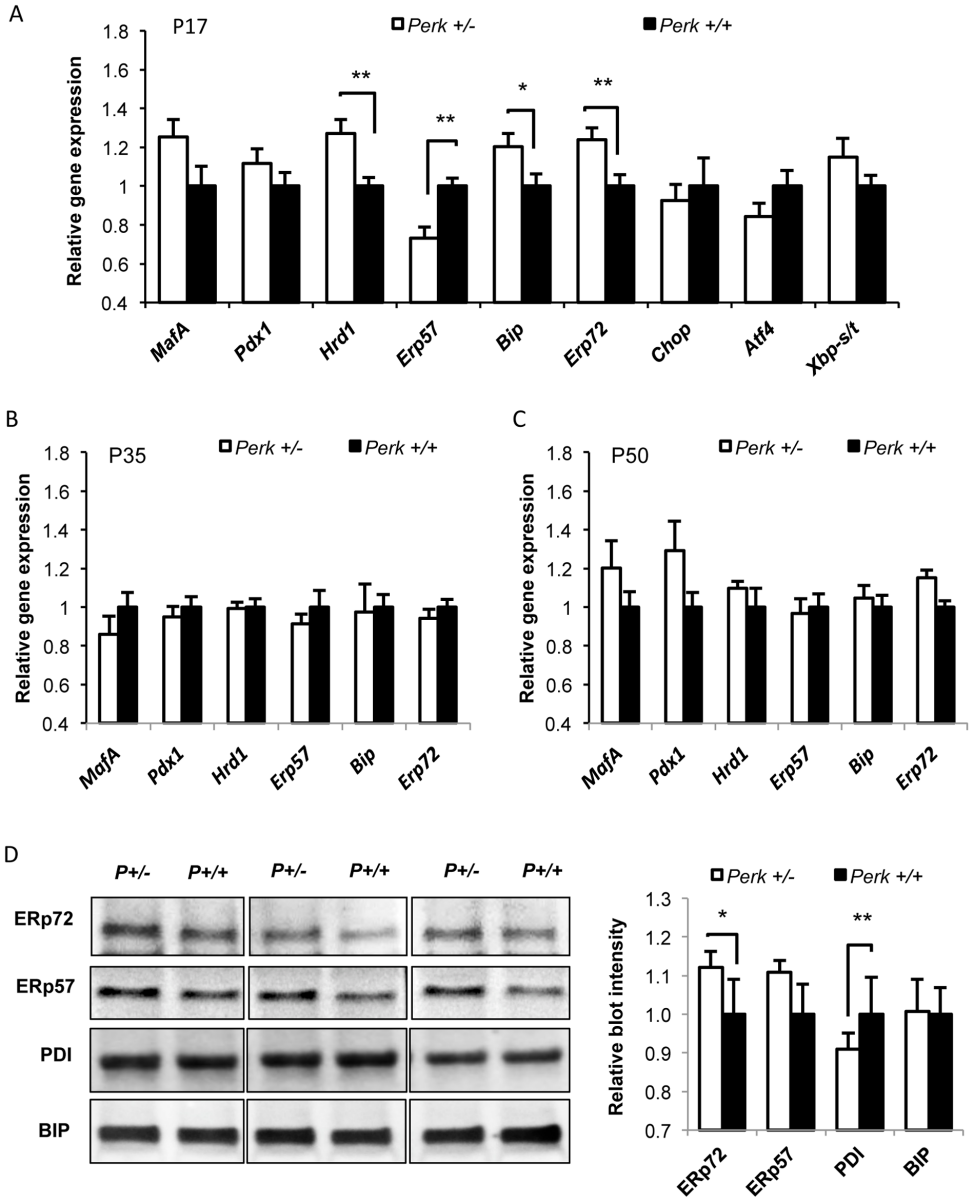
ER chaperones were impacted in P17 *Perk^+/−^* mice. A–C. Gene expression levels of mouse islets relative to *Perk^+/+^*. Mice are postnatal 17 days (figure A, n = 11,9), 35 days (figure B, n = 8, 8), and 50 days (figure C, n = 4,3). Shown are means ± SEM. (*P<0.05, **P<0.01). D. Western blot analysis of islets isolated from P17 mice. Left panel showed representative blots. Right panel showed quantification of blot intensity in relative to *Perk^+/+^*. (n for *Perk ^+/−^, Perk^+/+^*  = 5, 5. *P<0.05, **P<0.01).

To determine if the expression of other genes associated with insulin biosynthesis exhibited *Perk* genotypic differences in mice at P17, mRNA levels were determined in isolated islets for *MafA, Pdx1, Hrd1, ERp57, BiP,* and *ERp72*. *MafA* mRNA was increased by 25% (p = 0.06) in *Perk^+/−^* whereas P*dx1* was not changed (Fig 7A). The expression of the mRNAs encoding the ER chaperones HRD1, BIP, and ERp72 levels were significantly elevated in *Perk^+/−^* β-cells, while *ERp57* mRNA was reduced (Fig 7A). The expression of the same genes was measured in isolated islets of mice at P30 (Fig 7B) and P50 (Fig 7C) but none showed a genotypic difference. The protein levels of BIP, ERp72, ERp57 and another protein disulfide isomerase family protein PDI were assessed in pancreatic islets of P17 mice. ERp72 was elevated *Perk^+/−^* islets (Fig 7D), whereas PDI was decreased and BIP and ERp57 were not different from levels seen in *Perk^+/+^* islets. In addition, we also measured mRNA level of *Chop, Atf4 and Xbp-1* splicing in P17 mouse islets, which are sensitive indicators of ER stress. None of the ER stress markers showed *Perk* genotypic differences (Fig 7A), suggesting that regulation of β-cell functions by *Perk* dosage was not mediated through ER stress pathway.

### *Perk* gene dosage specifically in the pancreatic β-cells regulates glucose homeostasis

**Figure 8:**
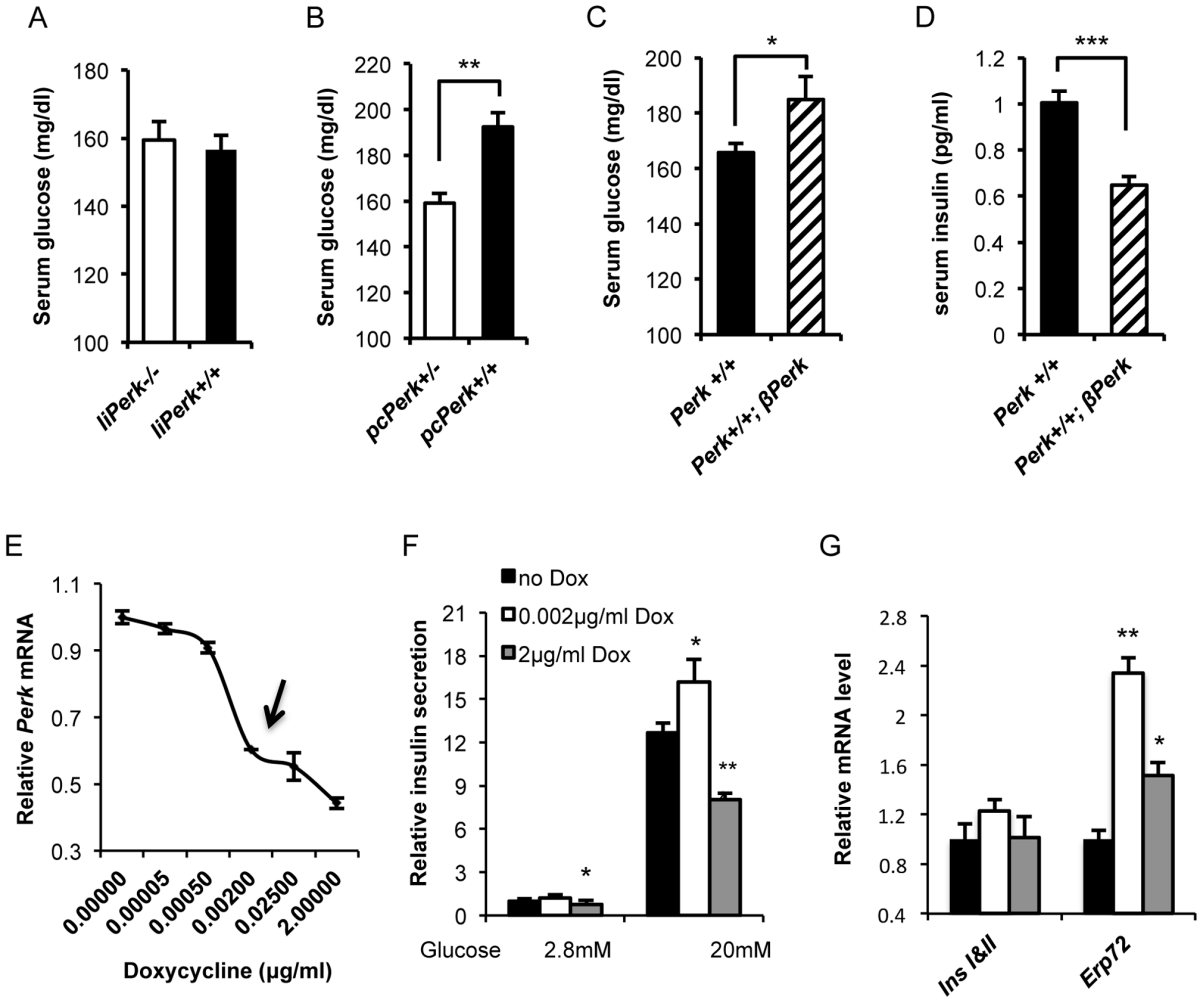
*Perk* gene dosage specifically in the β-cells regulates glucose homeostasis and insulin secretion. A. Random fed serum glucose of liver-specific *Perk* KO mice (*liPerk^−/−^)* or *liPerk^+/+^* at 70 days old. (n = 8, 8. A trend towards high blood glucose was seen in *liPerk^−/−^* mice but was not significantly different from wild-type control mice.). B. Random fed serum glucose of pancreatic-specific *Perk^+/−^ (pcPerk^+/−^)* or *pcPerk^+/+^* at age of P17-P35. Mice are in C57B/L6 background (n for *pcPerk^+/−^*, *pcPerk^+/+^*  = 18, 43. **P<0.01). C. Random fed serum glucose of *Perk^+/+^;βPerk* mice or wild-type littermates at age of P21-P38. Mice are in C57B/L6 background (n for *Perk^+/+^*, *Perk^+/+^;βPerk*  = 63, 36. *P<0.05). D. Random fed serum insulin of *Perk^+/+^;βPerk* mice or wild-type littermates at age of P27-P64. Serum insulin of each mouse was normalized to the average of wild-type littermates. Mice are in C57B/L6 background (n for *Perk^+/+^*, *Perk^+/+^;βPerk*  = 44, 16. ***P<0.001). E. *Perk* mRNA measurement in *INS1* 832/13 *shPerk* β-cells pre-treated with indicated concentration of doxycycline for 24 hours. *Perk* mRNA level was normalized to the level of non-doxycycline control (n = 4 per dosage point). F. Insulin secretion in response to 2.8 mM or 20 mM glucose in *INS1* 832/13 *shPerk* β-cells pre-treated with indicated concentration of doxycycline for 24 hours. Insulin secretion was normalized to total protein and expressed as fold increase in relative to basal insulin secretion (2.8 mM glucose) of non-doxycycline control. Shown are means ± SEM. (n≥4 per treatment. Statistical significance was determined by comparing to no-doxycycline control. * P<0.05, *** P<0.001). G. Gene expression measurement in *INS1* 832/13 *shPerk* β-cells pre-treated with indicated concentration of doxycycline for 24 hours. The mRNA level of each gene was normalized to the level of no-doxycycline control (n = 4 per dosage point. Statistical significance was determined by comparing to no-doxycycline control. *P<0.05, **P<0.01).

Although our analysis of β-cell functions suggests that the *Perk* genotypic differences in glucose homeostasis are due to differences in expression levels of PERK in β-cells, other organs that are known to regulate glucose homeostasis, including the liver, may also participate in this regulation. To pinpoint the responsible organ/cell type, we generated mouse strains in which *Perk* gene dosage was altered in specific organs and/or cell types. Examination of liver specific *Perk KO* (*liPKO*) mice revealed no differences in random fed glucose levels (Fig 8A). By contrast, we previously reported that pancreatic specific *Perk KO (pcPKO)* rapidly developed severe hyperglycemia similar to global *Perk KO* mice [14]. In addition, we now report that *pcPKO* heterozygotes exhibit 21% (P<0.01) lower random fed glucose levels than corresponding wild-type control in mice 3–5 weeks old (Fig 8B), suggesting that reducing *Perk* gene dosage in half specifically in the pancreas recapitulates the reduced serum blood glucose seen in the *Perk* heterozygous mice. Consistent with these observations, mice expressing an extra copy of *Perk* specifically in β-cells with an otherwise wild-type background (*Perk^+/+^;βPerk*) exhibited significantly elevated serum glucose (P<0.05, Fig 8C) and reduced serum insulin (P<0.001, Fig 8D). Therefore, the effect of *Perk* gene dosage on insulin and glucose homeostasis is likely to be β∼cell specific.

We sought to reduce *Perk* expression in cultured β-cells to confirm the importance of *Perk* gene dosage in β-cells and the difference in insulin synthesis and secretion we observed in *Perk^+/−^* mice. To accomplish this we modulated *Perk* mRNA in *INS1 832/13* β-cells through regulating the expression of a stably integrated *shPerk* transgene under the control of doxycycline (denoted as *INS1* 832/13 *shPerk* cells). After 24-hour administration of various concentration of doxycycline ranging from 0 to 2 µg/ml, *Perk* mRNA level was modulated within a range of 39.7%–100% of normal (Fig 8E). Maximum knockdown of *Perk* mRNA was achieved by using 2 µg/ml doxycycline. After 24-hour treatment of 2 µg/ml doxycycline, cells exhibited impaired GSIS and significantly elevated *ERp72* expression (Fig 8F and 8G), which were consistent with previous observations in mice or culture cells with total ablation of PERK by other means [14], [36]. By examining the dose-response curve, we found that the application of 0.002 µg/ml doxycycline for 24 hours provided a 40% reduction in *Perk* mRNA (P<0.001, Fig 8E) that mimicked the levels observed in *Perk^+/−^* mice (Fig 1B). Using this strategy, we found that both glucose stimulated insulin secretion and *ERp72* gene expression were significantly elevated in *shPerk* cells treated with 0.002 µg/ml doxycycline for 24 hours (Fig 8F and 8G), which was consistent with our observations in *Perk^+/−^* β-cells.

## Discussion

A complete deficiency of PERK results in the severest form of insulin-dependent diabetes [12], [15], [19], and therefore we expected that *Perk* heterozygosity would either be recessive with no effect on glucose homeostasis or would be semi-dominant with reduced insulin and elevated blood glucose. Unexpectedly, we found that *Perk* heterozygous mice exhibit an over-dominant phenotype in early postnatal development characterized by elevated insulin and correspondingly reduced blood glucose levels and increased glucose clearance. By using tissue or cell specific *Perk* KO mice and β-cell targeted *Perk* transgene we previously demonstrated that the insulin insufficiency and complete loss of glucose homeostasis was caused by the absence of PERK in the β-cells. Using one of these strains we generated pancreatic-specific *Perk* heterozygotes and found that *pcPerk^+/−^* mice had reduced blood glucose similar to *Perk^+/−^*. Once again, this was opposite to what we expected based on the diabetic phenotype of *pcPerk^+/−^*mice. Previously we showed that wild-type *Perk* transgene exclusively targeted to be expressed in β-cells could reverse the diabetes of the *Perk* KO mouse [14], [27]. However, when this transgene is present in an otherwise wild-type (*Perk^+/+^*) background it results in the reduction of serum insulin and the elevation of blood glucose. Thus, circulating insulin and blood glucose levels are negatively and positively correlated, respectively, with *Perk* gene dosage in the pancreatic β-cells. The effect of *Perk* gene dosage on glucose homeostasis is amplified in combination with the dominant Akita insulin mutant, which progressively develops diabetes postnatally. Lower *Perk* gene dosage slows the progression of diabetes in the Akita mouse whereas overexpression of *Perk* specifically in β-cells hasten it [20]. The stark exception to this rule is when *Perk* gene dosage is equal to zero.

The expression of PERK in the liver has been suggested to play an important role in glucose homeostasis in the first few days of life when gluconeogenesis plays a crucial role in providing glucose to the neonates [15], [23]. However, we found that glucose homeostasis was unaffected by genetically deleting the *Perk* gene in the adult liver. Consequently we assert that the effect of *Perk* gene dosage on insulin and glucose homeostasis is unlikely to be dependent upon liver functions and is only dependent upon the relative expression of *Perk* in the insulin-secreting β-cells as also supported by direct manipulation of *Perk* gene dosage therein.

**Figure 9:**
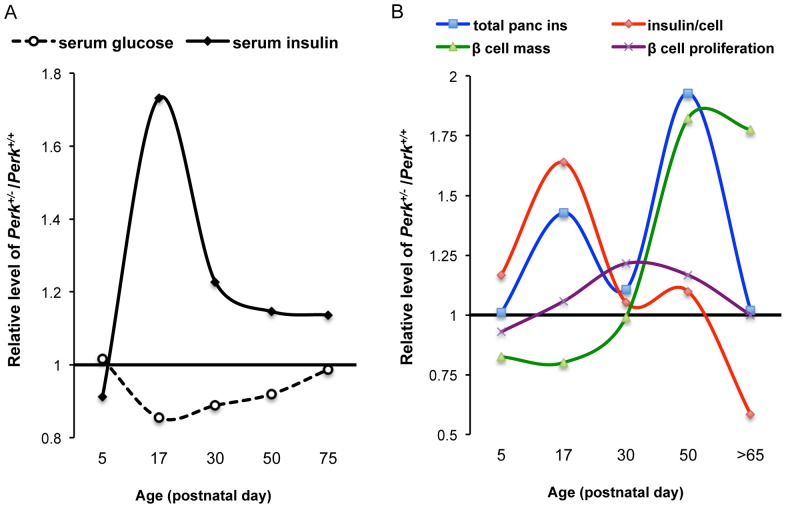
Developmental summary and model of PERK-dependent regulation of β-cell development and glucose homeostasis. A. Genotypic difference of serum glucose and insulin throughout development. Figure 9A was generated based on the data in Fig 2A and Fig 2C. Serum glucose and insulin are inversely correlated during postnatal development of *Perk^+/−^* mice. B. Genotypic difference of total pancreatic insulin content (based on data in Fig 3A), insulin content per β-cell (Fig 3C), β-cell number (Fig 3D), and β-cell proliferation rate (Fig 7B). Initially β-cell number is relatively low in *Perk^+/−^*, but β-cell and total pancreatic insulin are high. In response to low β-cell number, β-cell proliferation is accelerated between postnatal 17–50 resulting in increased β-cell number in *Perk^+/−^*. However, as β-cell number rises, insulin content per β-cell drops resulting in a balance between cell number and insulin concentration in each cell and a return to equivalent levels of total pancreatic insulin content.

Comparison of serum insulin and glucose levels throughout postnatal development shows a simple inverse relationship (Fig 9A). Given that we found no evidence for differences in peripheral insulin sensitivity before six-months of age, we conclude that *Perk* genotypic differences in blood glucose are directly determined by the amount of insulin secreted by the pancreatic β-cells. However the underlying reasons for elevated insulin secretion in *Perk* heterozygous mice change during postnatal development. Initially, as seen at postnatal day 17, total pancreatic insulin is elevated despite a reduced β-cell number indicating that each β-cell has substantially more stored insulin (Fig 9B). Later β-cell proliferation is accelerated in *Perk* heterozygotes. Although this acceleration is modest and transient, the compounding effect of increased proliferation over three weeks leads to a significant accrual of β-cell number in *Perk^+/−^* mice. The relative large β-cell number in *Perk* heterozygotes is maintained thereafter. However, as β-cell number increases insulin content per β-cell drops resulting in no genotypic difference in total pancreatic insulin in mice beyond 7 weeks. One constant observed across all ages is an elevation in the amount of insulin secreted per β-cell in *Perk* heterozygotes.

Recently we showed that PERK acutely regulates calcium dynamics and insulin secretion in human and rodent β-cells [25] independently of the eIF2α pathway. These experiments were performed using a PERK inhibitor that allowed us to determine the immediate effect of PERK on the crucial steps in calcium mobilization and insulin secretion. We concluded that PERK acts to regulate calcium dynamics and insulin secretion independently of its well-known role in phosphorylating the translation initiation factor eIF2α [25]. PERK has also been shown to regulate proinsulin quality control and trafficking in the endoplasmic reticulum [20], which is dependent on the phosphorylation of eIF2a by PERK. In the absence of PERK, proinsulin and ER client proteins eventually accumulate to extremely high levels in the ER and the ER ceases to function. The function of PERK in regulating ER quality control and trafficking is likely to be associated with its phosphorylation of eIF2α, as mutants of the regulatory phosphorylation site of eIF2α results in the same cellular phenotypes in β-cells [23]. These findings support the hypothesis that PERK has multiple functions in the pancreatic β-cells including immediate regulation of calcium dynamics and insulin secretion and long term regulation of the ER chaperones that orchestrate quality control, protein folding, and anterograde trafficking to distal compartments of the secretory pathway. In addition, PERK regulates β-cell proliferation as first demonstrated in *Perk* KO mice and now shown in *Perk* heterozygous mice [14]. It is unclear, whether the transient increase in β-cell proliferation in *Perk* heterozygotes is a primary function of PERK or is in response to changes in other β-cell functions.

The complex behavior of β-cells in *Perk* heterozygotes over the first few months of postnatal development can be subdivided into direct effects of PERK expression differences and adaptive responses to primary effects. Insulin secretion is likely to be a direct effect of PERK expression changes because it has been shown to be acutely regulated by PERK [25]. The relationship between insulin secretion and PERK expression exhibits a biphasic, inverted U-shaped dose response with half-dosage (*Perk^+/−^*) defining the maximum. This unexpected biphasic relationship between PERK expression and insulin secretion can account for the unexpected lower blood glucose levels seen in *Perk* heterozygotes. A similar inverted dose response relationship was noted between PERK expression and regulation of intermediary metabolism genes in a cell culture system [41]. More mysterious is the transient elevation of β-cell proliferation in *Perk* heterozygotes, which is unlikely to be due to a normal compensatory response in response to hyperglycemia and an increase demand for insulin similar to that seen in the progression to type 2 diabetes [42]. Previous studies suggested that intracellular Ca^2+^ positively regulates β-cell proliferation [43]–[45]. Given the fact that elevated insulin secretion continues to be observed in P30 and P50 *Perk^+/−^* mice, the increased β-cell proliferation in P30 and P50 *Perk^+/−^* mice might be due PERK-dependent regulation of Ca^2+^ signaling and insulin secretion. Regardless of the reason, the enhanced β-cell proliferation results in an increase in β-cell mass in *Perk^+/−^* mice, which is maintained indefinitely in the mature adult. The decrease in stored insulin that is seen in older adult mice (> 2 months old) is strongly correlated with the increased β-cell mass and is therefore likely to be a compensatory response to avoid hyperinsulimia. Among the five postnatal stages studied, P17 exhibited the largest array of differences including insulin gene transcription, proinsulin synthesis, insulin content, insulin secretion, hypoglycemia, β-cell mass, and changes in the expression of ER chaperones. It is likely that subsequent changes represent adaptive response in order to maintain glucose homeostasis.

Harding and Ron [12] independently generated a *Perk* KO mouse, which exhibited the same phenotype as our *Perk* KO strain including diabetes. Harding and Ron reported that older *Perk* heterozygous mice (6 months old) exhibited mild glucose intolerance in three different genetic backgrounds and this defect was stable from 6 months to 1 year old [12]. Although our *Perk* heterozygous mice show the opposite phenotype in terms of glucose tolerance in younger mice, they exhibit mild glucose intolerance at 6 months of age (data not shown). Moreover, the highly significant difference in random fed glucose levels between *Perk* genotypes observed in younger mice gradually diminishes with age and becomes non-significant. Taken together we speculate that the higher level of circulating insulin that we observed in *Perk* heterozygous mice during early postnatal development eventually lead to insulin resistance in older mice analogous to the progression of type 2 diabetes from compensation to de-compensation [42].

In conclusion, the complex and dynamic regulation of β-cell functions revealed by investigating *Perk* heterozygotes strongly argues that PERK has important physiological and postnatal developmental functions in β-cells. These functions are not likely to entail the well-known function of PERK to regulate the ER stress response in cultured cells as markers for ER stress were not differentially expressed.

## References

[1] Takane KK, Kleinberger JW, Salim FG, Fiaschi-Taesch NM, Stewart AF (2012) Regulated and reversible induction of adult human beta-cell replication. Diabetes 61: 418–424. 22210317 10.2337/db11-0580 PMC3266420

[2] Cozar-Castellano I, Fiaschi-Taesch N, Bigatel TA, Takane KK, Garcia-Ocana A, et al (2006) Molecular control of cell cycle progression in the pancreatic beta-cell. Endocr Rev 27: 356–370. 16638909 10.1210/er.2006-0004

[3] Weir GC, Bonner-Weir S (2013) Islet beta cell mass in diabetes and how it relates to function, birth, and death. Ann N Y Acad Sci 1281: 92–105. 23363033 10.1111/nyas.12031 PMC3618572

[4] Kushner JA (2013) The role of aging upon beta cell turnover. J Clin Invest 123: 990–995. 23454762 10.1172/JCI64095 PMC3582123

[5] Rorsman P, Renstrom E (2003) Insulin granule dynamics in pancreatic beta cells. Diabetologia 46: 1029–1045. 12879249 10.1007/s00125-003-1153-1

[6] Straub SG, Sharp GW (2002) Glucose-stimulated signaling pathways in biphasic insulin secretion. Diabetes Metab Res Rev 18: 451–463. 12469359 10.1002/dmrr.329

[7] Uchizono Y, Alarcon C, Wicksteed BL, Marsh BJ, Rhodes CJ (2007) The balance between proinsulin biosynthesis and insulin secretion: where can imbalance lead? Diabetes Obes Metab 9 Suppl 256–66. 17919179 10.1111/j.1463-1326.2007.00774.x

[8] Kane C, Shepherd RM, Squires PE, Johnson PR, James RF, et al (1996) Loss of functional KATP channels in pancreatic beta-cells causes persistent hyperinsulinemic hypoglycemia of infancy. Nat Med 2: 1344–1347. 8946833 10.1038/nm1296-1344

[9] Artner I, Hang Y, Mazur M, Yamamoto T, Guo M, et al (2010) MafA and MafB regulate genes critical to beta-cells in a unique temporal manner. Diabetes 59: 2530–2539. 20627934 10.2337/db10-0190 PMC3279542

[10] Greeley SA, Naylor RN, Philipson LH, Bell GI (2011) Neonatal diabetes: an expanding list of genes allows for improved diagnosis and treatment. Curr Diab Rep 11: 519–532. 21993633 10.1007/s11892-011-0234-7 PMC3226065

[11] Aguilar-Bryan L, Bryan J (2008) Neonatal diabetes mellitus. Endocr Rev 29: 265–291. 18436707 10.1210/er.2007-0029 PMC2528857

[12] Harding HP, Zeng H, Zhang Y, Jungries R, Chung P, et al (2001) Diabetes mellitus and exocrine pancreatic dysfunction in perk-/- mice reveals a role for translational control in secretory cell survival. Mol Cell 7: 1153–1163. 11430819 10.1016/s1097-2765(01)00264-7

[13] Cavener DR, Gupta S, McGrath BC (2010) PERK in beta cell biology and insulin biogenesis. Trends Endocrinol Metab 21: 714–721. 20850340 10.1016/j.tem.2010.08.005 PMC2991375

[14] Zhang W, Feng D, Li Y, Iida K, McGrath B, et al (2006) PERK EIF2AK3 control of pancreatic beta cell differentiation and proliferation is required for postnatal glucose homeostasis. Cell Metab 4: 491–497. 17141632 10.1016/j.cmet.2006.11.002

[15] Zhang P, McGrath B, Li S, Frank A, Zambito F, et al (2002) The PERK eukaryotic initiation factor 2 alpha kinase is required for the development of the skeletal system, postnatal growth, and the function and viability of the pancreas. Mol Cell Biol 22: 3864–3874. 11997520 10.1128/MCB.22.11.3864-3874.2002 PMC133833

[16] Harding HP, Novoa I, Zhang Y, Zeng H, Wek R, et al (2000) Regulated translation initiation controls stress-induced gene expression in mammalian cells. Mol Cell 6: 1099–1108. 11106749 10.1016/s1097-2765(00)00108-8

[17] Harding HP, Zhang Y, Ron D (1999) Protein translation and folding are coupled by an endoplasmic-reticulum-resident kinase. Nature 397: 271–274. 9930704 10.1038/16729

[18] Shi Y, Vattem KM, Sood R, An J, Liang J, et al (1998) Identification and characterization of pancreatic eukaryotic initiation factor 2 alpha-subunit kinase, PEK, involved in translational control. Mol Cell Biol 18: 7499–7509. 9819435 10.1128/mcb.18.12.7499 PMC109330

[19] Delepine M, Nicolino M, Barrett T, Golamaully M, Lathrop GM, et al (2000) EIF2AK3, encoding translation initiation factor 2-alpha kinase 3, is mutated in patients with Wolcott-Rallison syndrome. Nat Genet 25: 406–409. 10932183 10.1038/78085

[20] Gupta S, McGrath B, Cavener DR (2010) PERK (EIF2AK3) regulates proinsulin trafficking and quality control in the secretory pathway. Diabetes 59: 1937–1947. 20530744 10.2337/db09-1064 PMC2911049

[21] Iwawaki T, Akai R, Kohno K (2010) IRE1alpha disruption causes histological abnormality of exocrine tissues, increase of blood glucose level, and decrease of serum immunoglobulin level. PLoS One 5: e13052. 20885949 10.1371/journal.pone.0013052 PMC2946364

[22] Usui M, Yamaguchi S, Tanji Y, Tominaga R, Ishigaki Y, et al (2012) Atf6alpha-null mice are glucose intolerant due to pancreatic beta-cell failure on a high-fat diet but partially resistant to diet-induced insulin resistance. Metabolism 61: 1118–1128. 22386934 10.1016/j.metabol.2012.01.004

[23] Scheuner D, Song B, McEwen E, Liu C, Laybutt R, et al (2001) Translational control is required for the unfolded protein response and in vivo glucose homeostasis. Mol Cell 7: 1165–1176. 11430820 10.1016/s1097-2765(01)00265-9

[24] Back SH, Scheuner D, Han J, Song B, Ribick M, et al (2009) Translation attenuation through eIF2alpha phosphorylation prevents oxidative stress and maintains the differentiated state in beta cells. Cell Metab 10: 13–26. 19583950 10.1016/j.cmet.2009.06.002 PMC2742645

[25] Wang R, McGrath BC, Kopp RF, Roe MW, Tang X, et al (2013) Insulin secretion and Ca2+ dynamics in beta-cells are regulated by PERK (EIF2AK3) in concert with calcineurin. J Biol Chem 288: 33824–33836. 24114838 10.1074/jbc.M113.503664 PMC3837125

[26] Senee V, Vattem KM, Delepine M, Rainbow LA, Haton C, et al (2004) Wolcott-Rallison Syndrome: clinical, genetic, and functional study of EIF2AK3 mutations and suggestion of genetic heterogeneity. Diabetes 53: 1876–1883. 15220213 10.2337/diabetes.53.7.1876

[27] Li Y, Iida K, O'Neil J, Zhang P, Li S, et al (2003) PERK eIF2alpha kinase regulates neonatal growth by controlling the expression of circulating insulin-like growth factor-I derived from the liver. Endocrinology 144: 3505–3513. 12865332 10.1210/en.2003-0236

[28] Kitamura T, Kido Y, Nef S, Merenmies J, Parada LF, et al (2001) Preserved pancreatic beta-cell development and function in mice lacking the insulin receptor-related receptor. Mol Cell Biol 21: 5624–5630. 11463843 10.1128/MCB.21.16.5624-5630.2001 PMC87283

[29] Schmidt EK, Clavarino G, Ceppi M, Pierre P (2009) SUnSET, a nonradioactive method to monitor protein synthesis. Nat Methods 6: 275–277. 19305406 10.1038/nmeth.1314

[30] Gupta S, McGrath B, Cavener DR (2009) PERK regulates the proliferation and development of insulin-secreting beta-cell tumors in the endocrine pancreas of mice. PLoS One 4: e8008. 19956728 10.1371/journal.pone.0008008 PMC2776514

[31] Senee V, Chelala C, Duchatelet S, Feng D, Blanc H, et al (2006) Mutations in GLIS3 are responsible for a rare syndrome with neonatal diabetes mellitus and congenital hypothyroidism. Nat Genet 38: 682–687. 16715098 10.1038/ng1802

[32] Owen CR, Kumar R, Zhang P, McGrath BC, Cavener DR, et al (2005) PERK is responsible for the increased phosphorylation of eIF2alpha and the severe inhibition of protein synthesis after transient global brain ischemia. J Neurochem 94: 1235–1242. 16000157 10.1111/j.1471-4159.2005.03276.x

[33] Liang SH, Zhang W, McGrath BC, Zhang P, Cavener DR (2006) PERK (eIF2alpha kinase) is required to activate the stress-activated MAPKs and induce the expression of immediate-early genes upon disruption of ER calcium homoeostasis. Biochem J 393: 201–209. 16124869 10.1042/BJ20050374 PMC1383678

[34] Iida K, Li Y, McGrath BC, Frank A, Cavener DR (2007) PERK eIF2 alpha kinase is required to regulate the viability of the exocrine pancreas in mice. BMC Cell Biol 8: 38. 17727724 10.1186/1471-2121-8-38 PMC2072952

[35] Bobrovnikova-Marjon E, Hatzivassiliou G, Grigoriadou C, Romero M, Cavener DR, et al (2008) PERK-dependent regulation of lipogenesis during mouse mammary gland development and adipocyte differentiation. Proc Natl Acad Sci U S A 105: 16314–16319. 18852460 10.1073/pnas.0808517105 PMC2570995

[36] Feng D, Wei J, Gupta S, McGrath BC, Cavener DR (2009) Acute ablation of PERK results in ER dysfunctions followed by reduced insulin secretion and cell proliferation. BMC Cell Biol 10: 61. 19732428 10.1186/1471-2121-10-61 PMC2749809

[37] Trinh MA, Kaphzan H, Wek RC, Pierre P, Cavener DR, et al (2012) Brain-Specific Disruption of the eIF2alpha Kinase PERK Decreases ATF4 Expression and Impairs Behavioral Flexibility. Cell Rep 1: 676–688. 22813743 10.1016/j.celrep.2012.04.010 PMC3401382

[38] Zhang W, Feng D, Li Y, Iida K, McGrath B, et al (2006) PERK EIF2AK3 control of pancreatic beta cell differentiation and proliferation is required for postnatal glucose homeostasis. Cell Metabolism 4: 491–497. 17141632 10.1016/j.cmet.2006.11.002

[39] Senée V, Chelala C, Duchatelet S, Feng D, Blanc H, et al (2006) Mutations in GLIS3 are responsible for a rare syndrome with neonatal diabetes mellitus and congenital hypothyroidism. Nature Genetics 38: 682–687. 16715098 10.1038/ng1802

[40] Evans-Molina C, Garmey JC, Ketchum R, Brayman KL, Deng S, et al (2007) Glucose regulation of insulin gene transcription and pre-mRNA processing in human islets. Diabetes 56: 827–835. 17327454 10.2337/db06-1440 PMC3705758

[41] Oyadomari S, Harding HP, Zhang Y, Oyadomari M, Ron D (2008) Dephosphorylation of translation initiation factor 2alpha enhances glucose tolerance and attenuates hepatosteatosis in mice. Cell Metab 7: 520–532. 18522833 10.1016/j.cmet.2008.04.011 PMC2474721

[42] Weir GC, Bonner-Weir S (2004) Five stages of evolving beta-cell dysfunction during progression to diabetes. Diabetes 53 Suppl 3S16–21. 15561905 10.2337/diabetes.53.suppl_3.s16

[43] Heit JJ, Karnik SK, Kim SK (2006) Intrinsic regulators of pancreatic beta-cell proliferation. Annu Rev Cell Dev Biol 22: 311–338. 16824015 10.1146/annurev.cellbio.22.010305.104425

[44] Heit JJ, Apelqvist AA, Gu X, Winslow MM, Neilson JR, et al (2006) Calcineurin/NFAT signalling regulates pancreatic beta-cell growth and function. Nature 443: 345–349. 16988714 10.1038/nature05097

[45] Elghazi L, Balcazar N, Bernal-Mizrachi E (2006) Emerging role of protein kinase B/Akt signaling in pancreatic beta-cell mass and function. Int J Biochem Cell Biol 38: 157–163. 16213777 10.1016/j.biocel.2005.08.017

